# Intraguild Predation Dynamics in a Lake Ecosystem Based on a Coupled Hydrodynamic-Ecological Model: The Example of Lake Kinneret (Israel)

**DOI:** 10.3390/biology6020022

**Published:** 2017-03-29

**Authors:** Vardit Makler-Pick, Matthew R. Hipsey, Tamar Zohary, Yohay Carmel, Gideon Gal

**Affiliations:** 1 Oranim Academic College of Education, Kiryat Tivon 36006, Israel; 2 Aquatic Ecodynamics, UWA School of Agriculture and Environment, The University of Western Australia, 35 Stirling Highway, Crawley, Western Australia 6009, Australia; matt.hipsey@uwa.edu.au; 3 Yigal Allon Kinneret Limnological Laboratory, Israel Oceanographic and Limnological Research, Migdal 1495001, Israel; tamarz@ocean.org.il (T.Z.); gal@ocean.org.il (G.G.); 4 Faculty of Civil and Environmental Engineering, Technion-Israel Institute of Technology, Technion City, Haifa 32000, Israel; yohay@technion.ac.il

**Keywords:** intraguild predation (IGP), ecosystem modeling, lake Kinneret, DYCD-FISH, sustainable management, *Mirogrex terraesanctae*

## Abstract

The food web of Lake Kinneret contains intraguild predation (IGP). Predatory invertebrates and planktivorous fish both feed on herbivorous zooplankton, while the planktivorous fish also feed on the predatory invertebrates. In this study, a complex mechanistic hydrodynamic-ecological model, coupled to a bioenergetics-based fish population model (DYCD-FISH), was employed with the aim of revealing IGP dynamics. The results indicate that the predation pressure of predatory zooplankton on herbivorous zooplankton varies widely, depending on the season. At the time of its annual peak, it is 10–20 times higher than the fish predation pressure. When the number of fish was significantly higher, as occurs in the lake after atypical meteorological years, the effect was a shift from a bottom-up controlled ecosystem, to the top-down control of planktivorous fish and a significant reduction of predatory and herbivorous zooplankton biomass. Yet, seasonally, the decrease in predatory-zooplankton biomass was followed by a decrease in their predation pressure on herbivorous zooplankton, leading to an increase of herbivorous zooplankton biomass to an extent similar to the base level. The analysis demonstrates the emergence of non-equilibrium IGP dynamics due to intra-annual and inter-annual changes in the physico-chemical characteristics of the lake, and suggests that IGP dynamics should be considered in food web models in order to more accurately capture mass transfer and trophic interactions.

## 1. Introduction

### 1.1. Intraguild Predation

**Figure 1:**
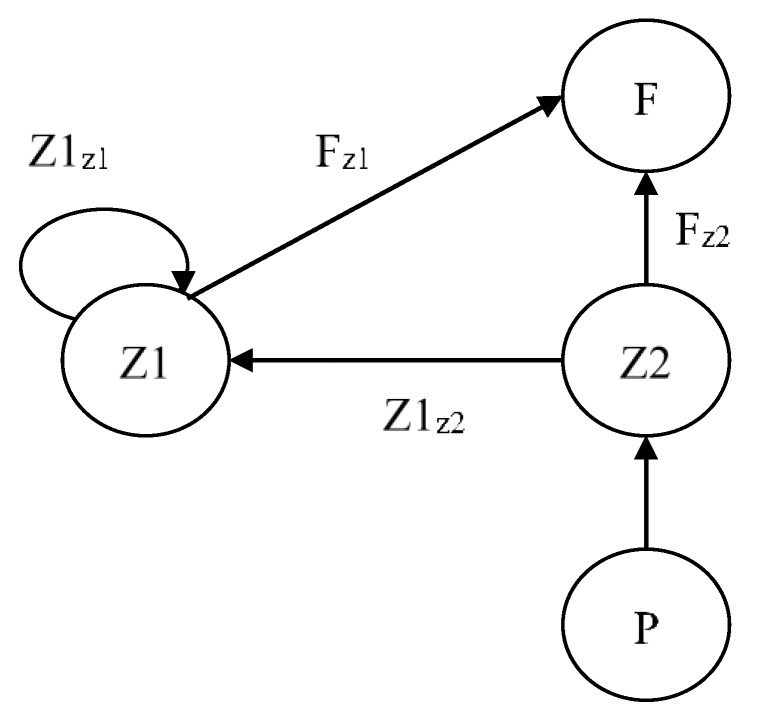
Schematic food web in an aquatic ecosystem containing an IGP component. F, Z1, Z2, and P, are fish, predatory zooplankton, herbivorous zooplankton, and phytoplankton, respectively. Fz1 and Fz2 are the fish predation rates for Z1 and Z2, respectively. Z1z1 and Z1z2 are Z1 predation rates for predatory and herbivorous zooplankton, respectively.

In many aquatic ecosystems, invertebrate predators such as predatory crustaceans (IG-prey) compete for food with the planktivorous fish (IG-predator), which, at the same time, may also prey on the predatory crustaceans, thereby creating an intraguild predation (IGP) relationship in the system [1,2,3] (Figure 1). IGP is probably more common in lakes than so far documented [4]. The theory predicts that productivity and the relative efficiency of resource (shared prey) utilization determine the outcome of interactions between IG-predators and IG-prey [2,5].

### 1.2. Modeling Intraguild Predation

To fully understand an ecosystem that includes IGP dynamics, a separate analysis of the IGP components and their impact on the ecosystem is necessary. This kind of analysis can be carried out using methods in which the abundance, distribution, diet, consumption rates, and predation pressure of the related IGP components are studied [6,7]. However, rigorous long-term empirical tests of equilibrium IGP interactions are rare [5,8,9,10], and long-term studies of IGP in natural systems are even scarcer [11,12], where conditions are far from equilibrium and influenced by external drivers. An alternative approach to explore food web dynamics that include IGP dynamics is by using numerical ecosystem models.

Food webs in which invertebrate predators are important cannot be modeled as a simple chain, as is regularly done in aquatic ecosystem models. Adding a compartment for invertebrate predators into lake food web models introduces an element of IGP [1,2,8]. It is also necessary to add an element of cannibalism/self-limitation, if observed in reality [13]. Such models are solved analytically and are used to explore ecological issues such as coexistence in IGP systems [14,15], alternative stable states [16], and the possibility of unstable dynamics, as well as problems in applied ecology such as the biological control of pest species, the transfer of pollutants through the pelagic food web [17], and the conservation of threatened species [18].

Although there is much variability among ecosystems, a known result of these models is that coexistence is feasible if the IG-prey is better than the IG-predator at competing for the shared prey, and if the IG-predator accrues a sufficient gain from attacking the IG-prey [1,19]. An IG-predator that is superior at suppressing the target herbivore population would stress an IG-prey population to extinction through a combination of competition and predation [1,19,20]. Additionally, Holt and Polis [1] showed that ecosystems with IGP have the potential to generate alternative stable states, with unstable dynamics that lead to transient phases. However, these models are based on several arguable assumptions, such as: (1) the system is assumed to be at equilibrium and the models are solved to equilibrium with constant meteorological forcing data [8,21]; or, (2) the two predators compete for only a single species of shared prey. Yet, according to Rosenheim and Harmon [22], the inclusion of multiple herbivore prey into IGP models excludes the prediction that the IGP is uniformly disruptive to biological control (i.e., the ability of the IGP-predator to suppress populations of herbivores); or, (3) the direct intraguild interactions are sufficiently common to be important for the IGP dynamics [23]. While simple models can often reproduce the elementary processes operating in complex systems and reveal fundamental ecological patterns [24], more complex mechanistic food web models are able to respond to the underlying variability in the physical-chemical conditions over a range of time-scales, and may better describe the natural feeding interactions [25]. A sufficient level of detail in IGP models is therefore essential for developing a deeper understanding of the predation rates and dynamics of zooplankton and fish, and their spatial and temporal variability [26]. However, to date, the explicit implementation and analysis of IGP in freshwater ecosystem models has been surprisingly rare.

### 1.3. Intraguild Predation in Lake Kinneret

The IGP component in the Lake Kinneret food web consists of the dominant fish in the lake, the zooplanktivorous *Mirogrex terraesanctae* [27], commonly known as Lavnun, and the predatory invertebrates (cyclopoid copepods); both feed on herbivorous zooplankton, while the fish also feed on the cyclopoid copepods. For more than two decades (1970–1993), the Lavnun constituted an important share of the Lake Kinneret commercial fishery, with a fairly constant catch of ~1000 t·y^−1^ [28]. In the winter of 1991/92, and again in 2002/03, the Lavnun fishery collapsed as the population became devoid of individuals of commercially harvestable sizes (>12 cm, [29]). This was in tandem with exceptionally high precipitation in the winter of 1991/92, and again in 2002/03, leading to >4 m rise in the lake’s water level, with major changes to littoral habitats where the Lavnun spawns. Hydroacoustic surveys indicated that the abundance of mainly sub-commercial sized Lavnun in Lake Kinneret in 1993, and again in 2004, increased by 8–10 fold in comparison to fish abundance levels prior to the flood years [30]. These exceptional increases in fish density were the consequence of the unusually successful recruitment of Lavnun as a result of the extreme water level increases. Hereafter, the term “atypical” will refer to an exceptionally high fish abundance (an abundance which is eight fold or higher than the multiannual average), as was observed approximately one to two years after an atypical high inflow winter.

During the 1980s and early 1990s, crustacean (predatory) zooplankton biomass dropped precipitously, reaching its lowest ever annual mean value (0.57 g_ww_·m^−3^) by 1993, in tandem with the collapse of the Lavnun fishery [31]. Furthermore, the predatory zooplankton underwent a regime shift due to the marked changes in abundance, evident as a gradual replacement of the larger-bodied *Mesocyclops* sp. with the smaller-bodied *Thermocyclops* sp. [32]. Gophen et al. [33,34] argued that Lake Kinneret demonstrates a top-down controlled system in which the Lavnun serves as the major predator of herbivorous zooplankton. Based on this assumption, a subsidized dilution program of the Lavnun was initiated as a management policy in 1995 and continued until 2006, whereby an annual amount of 400–500 t of commercial and sub-commercial size Lavnun fish were harvested. The objective of this subsidized harvest was to reduce the predation pressure on zooplankton, leading to an increase in their abundance and a reduction of phytoplankton, through grazing pressure, and ultimately, an improvement of the water quality. However, no correlation was found between the subsidized harvest and the Kinneret Water Quality Index [35] or other water quality indicators, suggesting that top-down control was not occurring as hypothesized.

Blumenshine and Hambright [6] compared the potential predation pressure on Lake Kinneret herbivorous zooplankton by Lavnun with that of the cyclopoid copepods. Despite having a much lower biomass than Lavnun, cyclopoid copepods accounted for a greater portion of the predation mortality of herbivorous zooplankton. This suggested that the intentional removal of Lavnun would not result in a subsequent increase in herbivorous zooplankton biomass as expected according to top-down theory. Instead, a reduction in Lavnun predation pressure may allow for an increase in the abundance of cyclopoid copepod and thereby result in a net increase in the predation pressure on herbivorous zooplankton.

A food web model developed for Lake Kinneret by Hart et al. [36] also predicted that planktivorous fish may serve as minor predators of herbivorous zooplankton, with the majority of its top-down regulation associated with cyclopoid copepods. Hart [13] explored the effects of IGP and self-limiting factors on the top-down and bottom-up properties of Lake Kinneret using the different models constructed based on the Lake Kinneret carbon flux model. The results indicated that IGP can reduce or even reverse the top-down effects predicted by the food chain theory, as a decrease in planktivorous fish is accompanied by an increase in the predation of zooplankton on invertebrates, and that the degree of self-limitation among the IG-prey is a key factor in determining the direction and strength of the top-down response.

Although models such as Hart et al. [36] and Hart [13] extended our understanding of ecosystems that have an IGP component, they are limited in their ability to reveal insights into the details of IGP variability. In particular, we are interested in understanding how these IGP-dynamics respond to a highly variable physico-chemical environment and the associated complex patterns of planktonic succession [36,37,38,39,40,41]. Moreover, Roelke et al. [42] analyzed a long-term data-set of Lake Kinneret within the framework of an alternative states model and revealed a possible complex triggering mechanism and system hysteresis (e.g., a change in a variable threshold where alternative states are possible, to a threshold where alternative states will no longer be possible). Gal and Anderson [32] identified the occurrence of a regime shift in the zooplankton population. Therefore, to explore the IGP relationship under the non-equilibrium conditions of Lake Kinneret, we applied a complex aquatic ecosystem model (DYCD-FISH). Unlike the models of Hart [13], that capture the most important first-order interactions by means of simple physics, DYCD-FISH is able to simulate the seasonal dynamics of vertical stratification and the most important chemical and biological ecosystem components. DYCD-FISH has previously been configured to include the IGP trophic triangle [43], accounting for both the bottom-up and top-down pathways shaping the seasonal dynamics of biogeochemical and ecological processes. However the intricacies of the IGP relationship, as described above, were not considered.

The aims of this study were therefore: (1) to improve our understanding of the predator-prey interactions between the dominant fish species in Lake Kinneret, *Mirogrex terraesanctae*, and zooplankton; (2) to study the sensitivity of the key factors controlling the IGP triangle (e.g., predation rate, feeding preference, and vulnerability to fish predation); (3) to compare IGP dynamics during “atypical” conditions, whereby an extreme increase in the fish population occurs, relative to “typical” conditions; and (4) to explore the likely impacts of fishery biomanipulation on fish and zooplankton biomass.

## 2. Materials and Methods

DYCD-FISH was validated for Lake Kinneret as previously described in Gal et al. [39] and Makler-Pick et al. [43]. The model is briefly described below.

### 2.1. Ecological Configuration

The mechanistic model DYCD-FISH was used to simulate the interactions between the hydrodynamics, biogeochemistry, bacteria, phytoplankton, zooplankton, and fish within Lake Kinneret.

DYRESM-CAEDYM (DYnamic REservoir Simulation Model-Computational Aquatic Ecosystem DYnamics Model, DYCD) is a one-dimensional coupled hydrodynamic-ecological model. DYRESM predicts the vertical distribution of temperature, salinity, and density, and models surface heat, mass and momentum transfers, mixed layer dynamics, hypolimnetic mixing, benthic boundary layer mixing, inflows, and outflows [44]. The model represents the lake as a series of homogeneous horizontal Lagrangian layers of variable thickness that expand or contract according to the degree of stratification and mixing, and the inflows or outflows entering or leaving the lake. CAEDYM is a generic aquatic ecological model in which a series of ordinary differential equations are solved to describe changes in the concentrations of nutrients (C, N, P, Si), detritus, dissolved oxygen, phytoplankton, and zooplankton, as a function of environmental forcing and ecological interactions for each layer represented by DYRESM. The variables of irradiance, temperature, salinity, and density are passed to CAEDYM, typically at a 1-h time step, and are used to determine the rates of change of biomass and chemical constituents for each of the ecological state variables.

DYCD-FISH couples a fish population model with DYCD to create a combined ecosystem-fish model capable of integrating the relatively slow metabolism of fish with other ecosystem components that often respond faster than fish. The model also accounts for the direct and indirect feedback between fish dynamics and the biogeochemical and planktonic modules. Fish directly affect the concentrations of the zooplankton, the particulate and dissolved organic matter (POM and DOM, respectively), oxygen, and the dissolved inorganic carbon in the different layers where the fish are located. Specifically, the Lake Kinneret version of DYCD-FISH is configured to simulate the carbon, nitrogen, phosphorus, and oxygen cycles, along with the biomass and metabolic processes of five phytoplankton groups (the dinoflagellate *Peridinium gatunense*; the filamentous diatom *Aulacoseira granulata*; the toxin-producing, N-fixing filamentous cyanobacterium *Aphanizomenon* sp.; the toxic colonial cyanobacterium that does not fix gaseous N, *Microcystis* sp.; and a general group of small-cell species, termed nanoplankton), three zooplankton groups (predatory zooplankton: adult stages of the predatory copepods and predatory rotifers; herbivorous zooplankton: cladocerans and copepodites; and micro-zooplankton including flagellates, ciliates, and copepod nauplii), heterotrophic bacteria (all simulated in units of mgC·L^−1^), and the population dynamics of the dominant fish in Lake Kinneret, *Mirogrex terraesanctae* [27].

The fish sub-model is based on a generic bioenergetics formulation, whereby an energy balance equation equates the energy gained as the difference between that consumed and metabolic expenses. Specifically, energy gain occurs through the difference between consumption and respiration, excretion, egestion, specific activity, and reproduction. The energy gained affects the growth rate and fish wet weight, and therefore, the growth rate of an individual fish is represented as the daily changes in wet weight per unit of fish weight per day (g_ww_·g_fish_^−1^·day^−1^).

In DYCD-FISH, the bioenergetics model is applied to many “super-individuals”, where each “super individual” represents numerous similar individuals [45], which each experience processes such as recruitment, and natural and fishing mortality that dynamically affect the number and biomass of fish. The population model takes advantage of the 1D hydrodynamic model, and at each time step, based on ambient conditions, the fish are redistributed in the water column with some stochastic variability, imitating the natural spatial movement and spread of the fish. Since the model is spatially (vertically) explicit, zooplankton, phytoplankton, and fish are located in varying water layers, such that the feeding intensity depends on the habitat overlap between predator and prey.

Additionally, an explicit recruitment model was developed to simulate the reported linkage between the change in water level and Lavnun spawning success. The specific parameters and customization used in the current fish population model are described in Makler-Pick et al. [43]. The fish-related variables examined in this study included fish biomass and the fish predation rate for zooplankton.

Since the focus of the manuscript is on fish-related food web dynamics associated with the IGP, we will now describe relevant components and interactions included in the Lake Kinneret implementation of DYCD-FISH. The Lavnun (the IG-predator) is an endemic, visually orienting zooplanktivore. As evident by the positive electivity values (i.e., preferences to food source) for Cladocera and Copepoda [46], it mainly feeds on Cladocera and Copepoda. The diet composition of the Lavnun also includes micro-zooplankton and particulate detrital material. When there are multiple prey types, the actual consumption depends on prey densities, the vulnerability of each prey item to the predator, the half-saturation constants governing the rate of feeding, and the maximum daily ration at a particular mass and temperature. Prey vulnerability, defined in the literature as the product of the encounter rate between predator and prey and the capture success of the predator [47], is dependent on several factors, such as abiotic conditions, the relative size of predator and prey, and predator activity and movements. Here, however, because of the lack of vulnerability experimental data, we used stomach content analysis [48,49] as an indicator of prey vulnerability and set the initial food vulnerability of the predatory zooplankton, herbivorous zooplankton, and micro-zooplankton to fish predation rates of 0.4, 0.5, and 0.02, respectively. The advantage of the current model is that it enables an exploration of the theoretical impacts of imprecise parameters, such as food vulnerability, the predation rate, and feeding preference, by substantially modifying their values. The diet of the predatory zooplankton, the IG-prey, consists of micro-zooplankton, herbivorous zooplankton, and the early stages of predatory zooplankton (via self-limiting predation) with preferences of 0.5, 0.35, 0.15, respectively. The main dietary source of herbivorous zooplankton (the IGP basal resource) is a multi-species group of small-celled phytoplankton termed the nanoplankton.

### 2.2. Model Base-Case Simulations

The simulations were configured to run from Jan. 1997 to Sep. 2003, at a 1 h time step for DYCD and a daily time step for the fish model. The initial conditions, forcing data, and input data are as described in Gal et al. [39] and Makler-Pick et al. [43]. The initial number of fish was set to 100 million fish in the lake (hereafter, ×1 or base level), as validated in Makler-Pick et al. [43]. The model time-series output files were used to study the temporal dynamics of zooplankton and fish biomass, as well as the predation rates of both fish and predatory zooplankton (the model is available upon request from the software developers). Multiple simulation runs verified that the stochastic component within the individual-based fish model resulted in negligible differences in fish population behavior for repeated simulation runs.

### 2.3. IGP Scenarios and Sensitivity Analysis

To simulate IGP dynamics when the number of fish is higher than the multiannual average, a series of scenarios were conducted, in which the number of fish represented by each “representative” super-individual was changed. The lake-wide fish population was initialized with 1000 “representative” fish, each set to represent 100,000 fish in the lake, equating to 100,000,000 fish in total. Then, this number was changed from an initial number of 100,000 (×1) to 200,000 (×2), and then up to 800,000 (×8) fish per “representative” fish, which was higher than the estimated increase in fish density recorded in 2004 [30], but enabled us to examine an extreme situation. Based on the outcome of the scenarios, we studied the impact of increasing fish numbers on the zooplankton biomass and predation rates. The results of complex models often suffer from limitations due to various sources of error and uncertainty, such as the initial conditions, input data, model structure, model parameters, and validation data [50]. However, a large source of model uncertainty is associated with the parameter values [50,51]. When a high uncertainty in the value of a parameter coincides with a high sensitivity of the model to that parameter, the reliability of the model predictions may be very low [52]. Makler et al. [53] and Gal et al. [54] evaluated the sensitivity of DYCD model parameters. Here, the sensitivity of the herbivorous zooplankton to different key factors playing a part in the IGP triangle are explicitly studied. The initial values of the following parameters: the maximum predation rate of the predatory zooplankton (g_max_, gC·m^−3^ (gZ·m^−3^)^−1^·day^−1^), the feeding preference of predatory zooplankton to the early stages of predatory zooplankton (cannibalism or self-limiting predation) (P_zk1_), and the vulnerability of predatory (V_11_) and herbivorous (V_12_) zooplankton to fish predation, were modified. The changes were made over the course of a series of scenarios by changing the initial value of each parameter, one at a time, by ±50%. The average concentration of the herbivorous zooplankton over the seven-year simulation was calculated and served as the basis for comparison.

### 2.4. Biomanipulation

Fish biomanipulation (namely, the positive size-selective harvest of mainly non-commercial size fish) is a possible management action aiming to control high fish recruitment, such as occurs in Lake Kinneret in atypical years, to reduce phytoplankton and ultimately improve the water quality. The size-selective harvest of the Lavnun was modeled by increasing the value of the fishing mortality (exploitation rate) parameter of non-commercial size fish from 0 to 50 (%·year^−1^), and the value of the fishing mortality parameter of commercial size fish from 28 to 50 (%·year^−1^), based on the scenario of an atypical year (×8 scenario).

## 3. Results

### 3.1. IGP Dynamics under Typical Conditions

**Figure 2:**
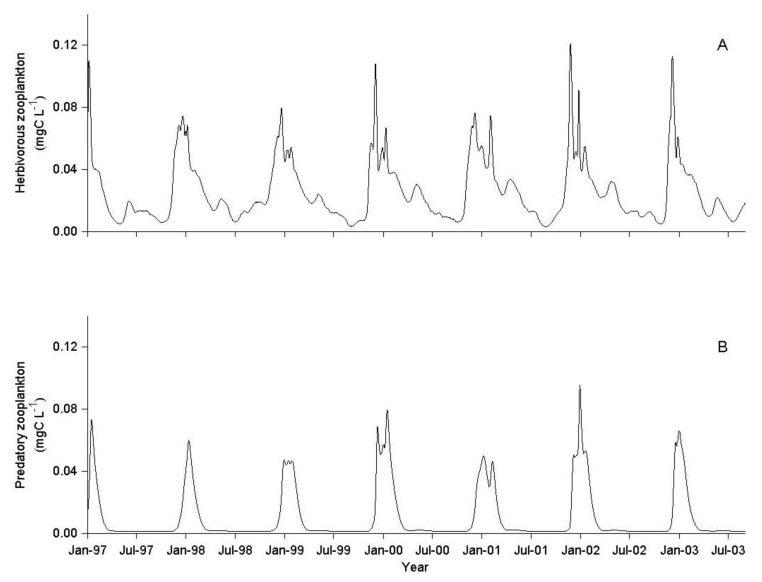
Simulation results (1/1/1997–1/9/2003) of (**A**) herbivorous zooplankton concentration (mgC·L^−1^) and (**B**) predatory zooplankton concentration (mgC·L^−1^) at base level of fish (×1).

The herbivorous zooplankton in the base simulation level (×1) showed seasonal biomass peaks from fall to spring, with low inter-annual variation throughout the simulation period (Figure 2A). The predatory zooplankton (cyclopoid copepods) showed annual biomass peaks in late winter to early spring, with low interim values (Figure 2B).

**Figure 3:**
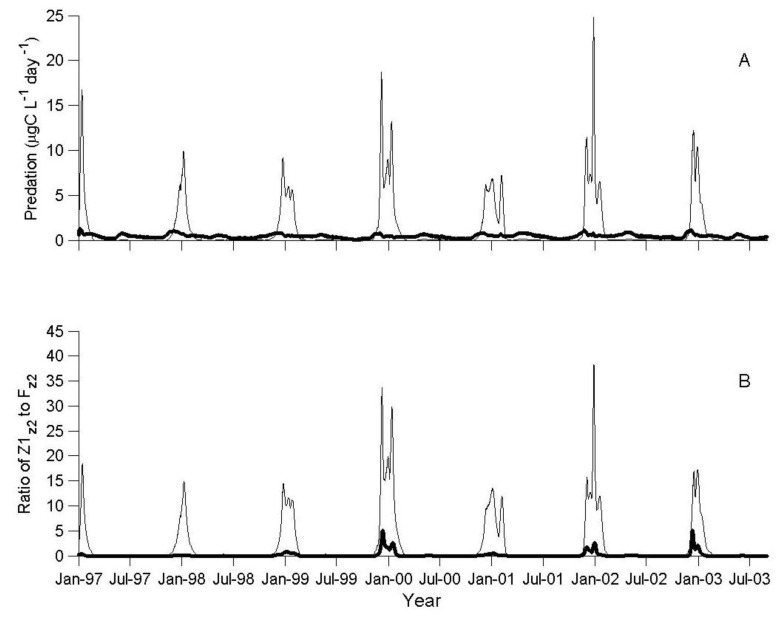
(**A**) Simulated predation rate (μgC·L^−1^·day^−1^) of predatory zooplankton (thin line) and fish (thick line) on herbivorous zooplankton; (**B**) Ratio of the zooplankton predation rate (Z1_z2_) to the fish-predation rate (F_z2_) for herbivorous zooplankton (Z2) at the base level of fish (thin line) and at an increased level of fish (×8, thick line).

The average simulated lake-wide total biomass of predatory cyclopoid over the years 1997–2003, was 799 × 10^6^ g_ww_, with a mean daily predation rate of 109 mg_prey_·g_pred_^−1^·day^−1^. During this period, the Lavnun had an average total lake biomass of 1281 × 10^6^ g_ww_ and the mean daily predation pressure on herbivorous zooplankton was 28 mg_prey_·g_pred_^−1^·day^−1^. The simulated predation pressure by both the Lavnun and the predatory zooplankton varied seasonally. During winter and early spring (November-February), predation by predatory zooplankton accounted for the majority of the predation mortality for herbivorous zooplankton (Figure 3A), imposing a predation pressure 10–20 times higher than the Lavnun predation pressure (Figure 3B).

**Figure 4:**
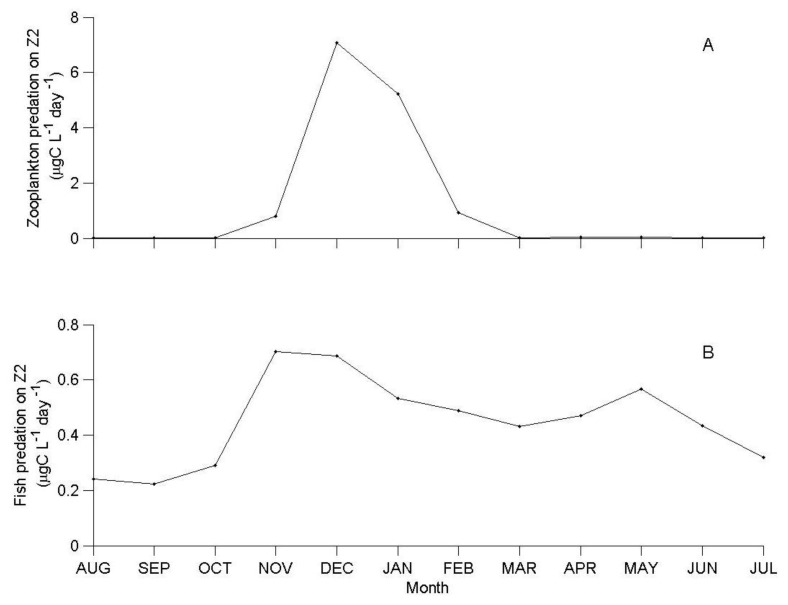
Simulated monthly average predation rate (μgC·L^−1^·day^−1^) of (**A**) predatory zooplankton and (**B**) fish, on herbivorous zooplankton (Z2) at base fish level (×1).

At the time of its annual peak, in December, the monthly average predation rate of predatory zooplankton was 7.0 μgC·L^−1^·day^−1^ (10% of herbivorous zooplankton biomass per day), and remained high in January, before declining to very low levels (0.004 μgC·L^−1^·day^−1^) between March and October (Figure 4A). During this time period, the fish predation rate was higher than the zooplankton predation rate (Figure 4B). The annual cycle of the fish predation rate was more moderate than that of the predatory zooplankton, ranging between a minimum of 0.2 μgC·L^−1^·day^−1^ in September, to a maximum of 0.7 μgC·L^−1^·day^−1^ in November (1% of herbivorous zooplankton biomass concentration per day).

**Figure 5:**
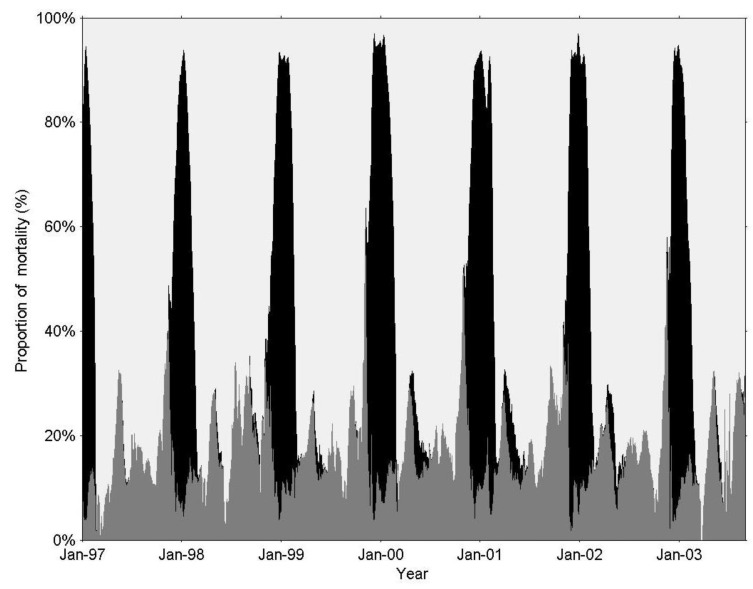
Simulated proportion (%) of total herbivorous zooplankton mortality due to non-predation mortality (light grey), zooplankton-predation (black), and fish-predation (dark grey).

From spring to autumn, fish predation was the dominant loss of herbivorous zooplankton, in tandem with a 40% lower herbivorous zooplankton biomass in comparison to late winter (November–February, Figure 2A and Figure 5). However, in late winter, during the short period of the annual biomass peak of the predatory zooplankton, there was an extremely high predation mortality (>80% of total herbivorous zooplankton) associated with zooplankton predation.

### 3.2. IGP Dynamics during High Fish Abundance

**Figure 6:**
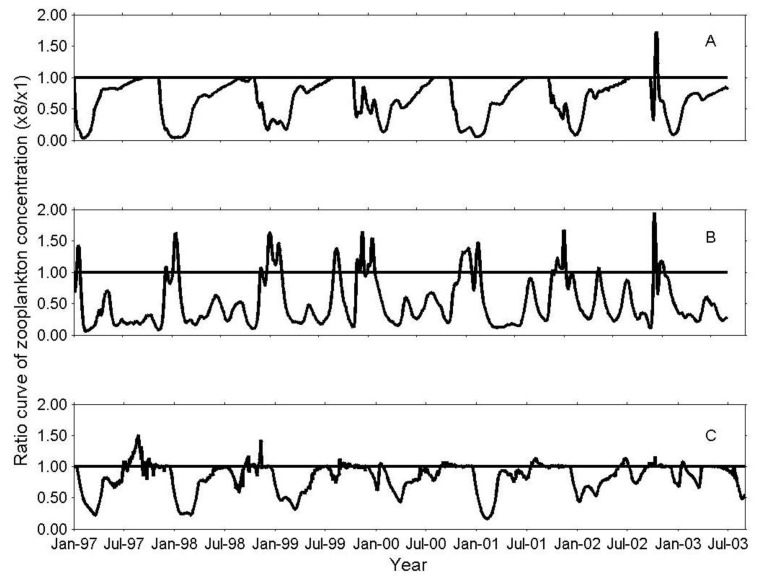
Ratio of zooplankton concentration at ×8 to zooplankton concentration at ×1 for (**A**) predatory zooplankton, (**B**) herbivorous zooplankton and (**C**) micro zooplankton.

Throughout most of the high fish abundance simulation (×8), the concentration of predatory zooplankton and micro-zooplankton was lower than in the base level scenario (ratio < 1 in Figure 6A,C). The herbivorous zooplankton biomass was also affected by the changes in fish densities. This impact, however, mainly occurred in the periods between the seasonal biomass peaks of the herbivorous zooplankton. In the presence of higher fish numbers, the concentration of herbivorous zooplankton during its simulated seasonal biomass peaks (January–February) was similar or even higher than the concentration simulated at the base level of fish. This phenomenon is indicated by values higher than one in Figure 6B.

In the presence of ×8 more fish, fish predation of herbivorous zooplankton dominates the predation in this group for most of the simulation period (values < 1 in Figure 3B), with some exceptions during the fourth, sixth, and seventh year of the simulation, when the ratio of zooplankton predation to fish predation was greater than one.

**Figure 7:**
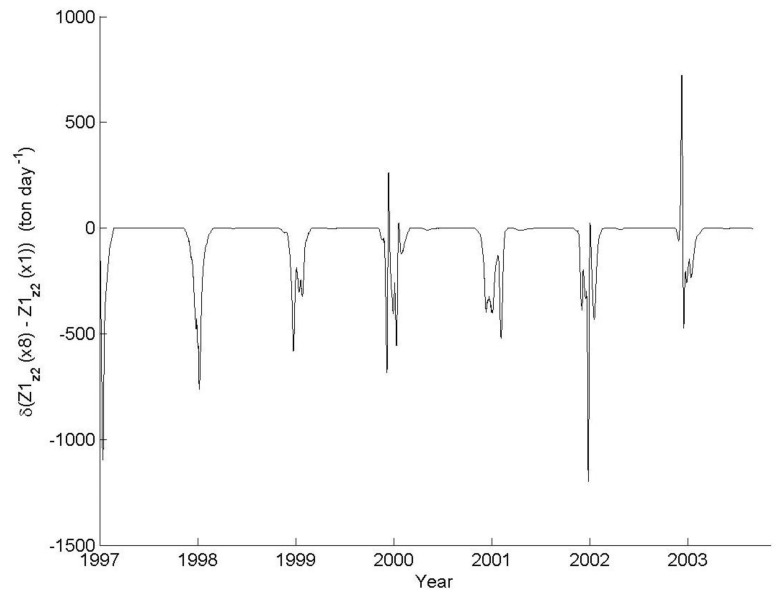
The difference between the predatory zooplankton (Z1) predation rate (on herbivorous zooplankton, Z2) at the base simulation (×1) and the ×8 scenario. Values represent the whole lake predation rates in ton day^−1^.

Substantial differences between the zooplankton predation pressure on herbivorous zooplankton in the ×8 scenario and the ×1 simulation are mainly observed during the annual peak of the herbivorous zooplankton biomass, demonstrating a considerable seasonal decrease in the net predation by predatory-zooplankton at the ×8 scenario (Figure 7). Intriguingly, the lower predation pressure may allow for an increase in herbivorous zooplankton biomass during these periods (Figure 6B). At all other times, model simulations suggest that the herbivorous zooplankton biomass is mainly controlled by fish predation.

The mean annual predation by fish and zooplankton on the different zooplankton groups at varying levels of fish abundance, are given in Table 1. At the base level (×1), the mean annual predation of predatory zooplankton is 2.5, 5.3, and 7.7 times higher than fish predation on herbivorous, predatory, and micro-zooplankton, respectively. These results demonstrate the predation superiority of the predatory zooplankton (over the fish) at the base level of fish. An increase in the number of fish resulted in a lower mean annual predation rate of predatory zooplankton, both on itself (i.e., self-limitation) and on the other zooplankton groups. The main impact was on the predatory zooplankton itself; the self-limitation was lowered to 9% of the base level in the presence of ×8 more fish. On average, the outcome of the ×8 scenario was a 70% lower total zooplankton predation (on all zooplankton groups), and on the other hand, a higher fish predation, on all its prey types (>200%). Interestingly, the total herbivorous zooplankton eaten annually, whether by fish or by zooplankton, was close to 44 × 10^3^ ton year^−1^ for all simulated scenarios, regardless of fish biomass. The total predation in the system (the sum of zooplankton predation and fish predation) ranged from 52 × 10^3^ to 60 × 10^3^ ton. At the base level of fish, and even when the number of fish was doubled, the ratio between the annual average of zooplankton predation to the annual average of fish predation on all zooplankton groups was greater than one, indicating that, in total, the main predator in the ecosystem is the predatory zooplankton (Table 1). A higher number of fish (×8) resulted in a ratio lower than one, indicating the dominance of fish in consuming zooplankton under these conditions.

### 3.3. The Effect of Biomanipulation

**Figure 8:**
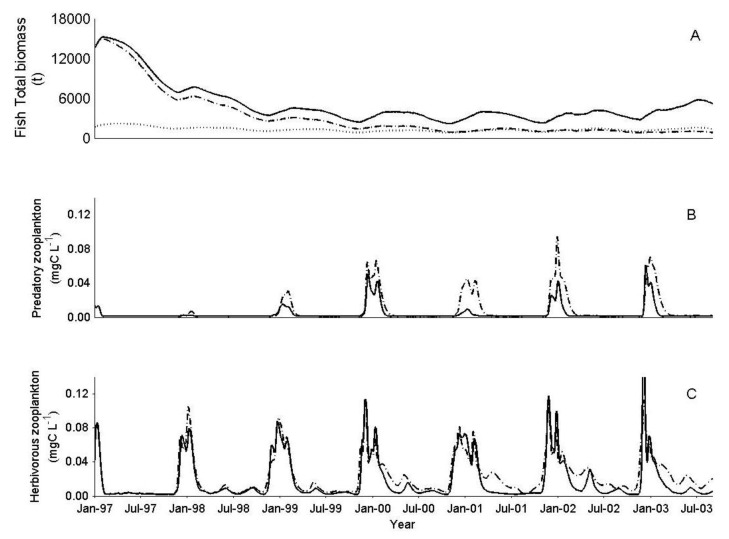
The effect of fishery biomanipulation (changing the commercial size fishing mortality parameter from 28 to 50 (%·year^−1^) and the non-commercial size fishing mortality parameter from 0 to 50 (%·year^−1^) on (**A**) total Lavnun biomass (t); (**B**) Predatory zooplankton concentration (mgC·L^−1^) and (**C**) herbivorous zooplankton concentration (mgC·L^−1^), before biomanipulation (solid line) and after biomanipulation (dashed line) at ×8 more fish, and, total Lavnun biomass at ×1 (dotted line).

Manipulating the fishery by imposing particularly heavy fishing mortality on non-commercial (<12 cm) and commercial size fish, at the ×8 scenario, demonstrated a decrease in the total fish biomass (Figure 8A) and an increase in the zooplankton biomass. The increase was particularly considerable for the predatory zooplankton (Figure 8B) and more moderate for the herbivorous zooplankton, where no substantial impact was shown in the timing of the annual peaks (Figure 8C).

### 3.4. Sensitivity Analysis of the IGP Component

The relative change (%) in comparison to the base model results is presented in Table 2. The results indicate that the greatest impact on the average herbivorous zooplankton biomass was by the maximum predation rate of the predatory zooplankton parameter (g_max_). Decreasing its value by 50% resulted in an increase of more than 27% in the herbivorous zooplankton biomass concentration, while increasing its value by 50% resulted in a 10% decrease in the herbivorous zooplankton concentration. Decreasing the vulnerability of herbivorous zooplankton to fish predation (V_12_) by 50% had a positive impact, resulting in a 10% increase in population size, and a comparable but negative impact was observed when the vulnerability was increased by 50% (−7%).

## 4. Discussion

The simulation results indicated that predatory zooplankton (and not the Lavnun) control herbivorous zooplankton predation when the abundance of fish is similar to the multi-annual average (Figure 3, Figure 4 and Figure 5); this accounts for a maximum daily mortality rate of 10% of the population, in comparison to 1% imposed by the fish. The predatory zooplankton consumption varied seasonally, and at the time of the annual biomass peaks it exerted a predation pressure that was 10–20 times higher than fish predation.

Predation rates are typically measured by feeding experiments with different food types and/or radioactively or fluorescently labeled food, serial dilution/concentration experiments, stable isotope experiments, gut content analyses, and bioenergetics modeling [55]. However, none of these methods are continuous, long-term, or in-situ, and therefore limit the validation of the model. Thus, to assess the simulation results, we compare several indirect metrics of the model simulation with available data from Lake Kinneret and other sites. For example, Blumenshine and Hambright [6], reported an order of magnitude higher mass specific consumption rate on Lake Kinneret herbivorous zooplankton by cyclopoid copepods than the Lavnun (mean value of 921 mg_prey_·g_pred_^−1^·day^−1^ for cyclopoid copepods in comparison to 74 mg_prey_·g_pred_^−1^·day^−1^ for Lavnun), and an exertion of 66% of the predation pressure on herbivorous zooplankton. In that research, fish consumption was estimated by using the observed Lavnun growth rate as the input for a bioenergetics model. The model output was an estimate of the amount of energy needed to manifest the observed growth rate. The monthly temperature-dependent specific ingestion rates for predatory zooplankton were based on data for adult female *M. ogunnus* from Gophen [56]. The difference between the simulation results in the current study and those reported by Blumenshine and Hambright [6], can be partly attributed to the different methods employed. For example, the DYCD model calculates zooplankton predation as the product of the predation rate coefficient, temperature dependence function of predation, and the overall potential rate of carbon consumption, which is regulated by a Michaelis-Menten function. Alternatively, Blumenshine and Hambright [6] calculated zooplankton predation rates based on the temperature-dependent empirical quadratic equation. Placing our model simulated data (i.e., simulated predation rate) and measured biomass of predatory zooplankton and herbivorous zooplankton into this equation, gives an average predatory zooplankton predation rate of 1029 mg_prey_·g_pred_^−1^·day^−1^, which is comparable to the 921 mg_prey_·g_pred_^−1^·day^−1^ reported by Blumenshine and Hambright [6], providing an additional indirect validation to DYCD-FISH.

In research conducted at the temperate Lake Ontario, Gal et al. [7] found that the consumption rates of *Mysis* (IG-prey) and fish (IG-predator) varied widely with the season, and that *Mysis* predation (2.6 × 10^−3^–1.3 g_prey_·m^−2^·day^−1^) is superior over fish predation (1.4 × 10^−3^–0.5 g_prey_·m^−2^·day^−1^) in the summer, but that fish predation is dominant in October. Morin [5] examined the interactions between two freshwater protists, *Colpidium striatum* (the IG-prey) and *Blepharisma americanum* (the IG-predator), in laboratory microcosm experiments. Their results supported the predictions of the IGP theory and the dominance of the IG-prey.

The outcome of exceptionally rapid and large increases in water levels in Lake Kinneret, and the subsequent increase in the number of Lavnun fish, can be described in terms of top-down control. The simulation results demonstrated that an eightfold increase of fish causes: (1) a shift of fish body size towards smaller sizes, resulting in most of the fish being of a sub-commercial size [57]; (2) lower zooplankton biomass (Figure 6); and (3) lower predation of zooplankton (Table 1). These results can be compared to reported data in the years 1993 and 2004 [58,59,60], in terms of the linkage between large changes in the water level, the following increase in the number of fish, and zooplankton abundance. Furthermore, these results support the hypothesis that large increases in fish abundance following extremely wet winters cause increased fish predation on zooplankton, thus drastically reducing the total zooplankton densities. Lake-based data from the years 1990–2004 confirm an exceptional decrease in the mean monthly densities, mainly of predatory zooplankton and herbivorous zooplankton one to two years after atypical wet years [32,58]. However, the decline is not consistent throughout the whole year; during certain months, zooplankton densities were high, relative to the levels observed in “typical” years. For example, in 2004, the observed annual average zooplankton density was one of the lowest ever recorded. Yet the density of herbivorous zooplankton in June was fairly high (70 organisms L^−1^) and the density of all zooplankton groups in that month was similar to the multi-annual mean [58,61]. The comparable results simulated in the ×8 more fish scenarios, serve not only as indirect validation of the model processes, but clearly illustrate the possible effects of an IGP component in the food web. For example, from December to February, in the presence of a higher number of fish (×8), the lower predation pressure by predatory zooplankton allows for a temporary higher biomass of herbivorous zooplankton in comparison to the ×1 fish scenario (Figure 6B). This finding illustrates how, in the presence of an extremely high number of fish, the system seasonally shifts the food web between being directly dominated by fish predation (when the food web somewhat resembles a linear food chain), to periods when the direct dominant effect, on herbivorous zooplankton, is the substantial release from the predatory zooplankton predation pressure caused by the increased fish predation.

The outcomes of the long-term (1994–2006) biomanipulation (removal of 400–500 ton fish year^−1^) employed in Lake Kinneret can be, at least partly, explained within the framework of this model and the complexities of IGP dynamics. In the first five years of the biomanipulation program, zooplankton abundance in Lake Kinneret increased, reaching a peak in the year 2000, similar to those measured in the 1970’s [31,58]. Since then, and disagreeing with the expectations, zooplankton abundance has steadily decreased, reaching one of its lowest ever mean annual biomass values in 2004 [58]. Simulating biomanipulation with DYCD-FISH showed: (1) a decrease in fish biomass; (2) an increase in predatory zooplankton biomass; and (3) a moderate increase in herbivorous zooplankton biomass in periods when predatory zooplankton biomass is relatively low and an insignificant change when predatory zooplankton is at its seasonal peak (Figure 8).

Blumenshine and Hambright [6] claim that the lower-than-expected impact of Lavnun removal on herbivorous zooplankton biomass in Lake Kinneret can be attributed to the possible boosting of cyclopoid copepod abundance, caused by the reduced Lavnun biomass, and the following increase in the net predation pressure on herbivorous zooplankton. The simulation results confirm this claim, interestingly also indicating that the total zooplankton loss to predation, and particularly that of herbivorous zooplankton, is largely indifferent to the amount of Lavnun. The specific predation on herbivorous zooplankton is relatively constant (44 × 10^3^ ton·year^−1^), but channeled differently into either predatory zooplankton or fish, depending on the fish density in the lake (Table 2). This was true not only for a small increase (×2) in the number of fish, but also for a substantial increase (×8). The simulated biomanipulation results suggest a possible reason why the culling program (the subsidized harvest) implemented in Lake Kinneret did not have the desired impact on the water quality. By removing fish from the lake (and decreasing fish predation pressure), zooplankton predation on herbivorous zooplankton is increased (mainly at the time of the seasonal peak), weakening the cascading effects of top-down control. This serves as an example for compensatory effects within the food web, where trophic interactions mediate resilience to the overall system in the sense that perturbations at the top of the food web are dampened and only marginally affect the lower parts of the food web [6]. The end result regarding the predation of herbivorous zooplankton is approximately the same, regardless of whether there are high or low numbers of fish.

The sensitivity test of the simulated herbivorous zooplankton biomass was conducted by changing the values of the grazing-related parameters, such as the “maximum predation rate of predatory zooplankton” (g_max_). Based on laboratory experiments [6,56] and model calibrations, the initial value of the parameter was set to 3.03 gC·m^−3^ (gZ·m^−3^)^−1^·day^−1^. The sensitivity analysis indicated that g_max_ had the highest impact on herbivore biomass, when compared with parameters such as “predatory zooplankton diet preference” and “zooplankton vulnerability to fish predation”. Our findings also indicate that increasing (or decreasing) g_max_ by 50% does not affect the seasonal predation dynamics or the conclusion, mentioned earlier, that a higher number of fish can seasonally reduce the predation pressure on the herbivorous zooplankton. According to the sensitivity test, the parameter “predatory zooplankton self-limitation” had a relatively low impact. It should be noted, however, that the self-limitation refers to the self-cannibalism of the predatory zooplankton, while in reality, it would be mainly due to adults consuming herbivorous copepodites or nauplii. According to Hart [13], the self-limitation among the IG-prey is a key factor in determining the direction and strength of the top-down response and the IG-predator will only induce an increase in the basal resource if IG-prey self-limitation is adequately strong.

Exploring IGP is challenging, both empirically and in terms of modelling. Many IGP systems are embedded in communities with several alternative prey species; therefore, there are often more species involved in intraguild predation than just the three used in the basic conceptual model. This can explain why simple theories and models fail to capture real-world data-sets [11,26]. As was demonstrated by a set of structured models [62], depending on the trophic position where self-limitation occurs, it can entirely modify the dynamics and structure of three-species IGP systems. Furthermore, empirical tests of ecological theory are limited by the mismatch between the short-term population dynamics data that are readily obtained from field experiments (i.e., changes in population size over one to at most several generations), and the long-term dynamics described by models (i.e., whether two species will coexist stably for many generations). The short duration of most field studies relative to the generation times of the organisms disrupt our ability to make strong conclusions about population stability or prolonged coexistence [5,11,63].

The difference between the DYCD-FISH and other models can be attributed to the more conceptual and theoretical nature of these models. Most IGP models described in the relevant literature are relatively simple [1,13,15,20]. The models employed by Hart [13], for example, were deliberately kept as simple as possible, so that they could be easily compared to simple food chain models. However, this approach is not able to account for major factors such as inedible algae, nutrient cycling, the nutrient limitation of consumers, and the seasonal dynamic interactions between all of these components and other food web components, making it unclear if they are able to induce dynamics which are different from those predicted by the classic food chain theory. In the current model, the IGP interactions are affected by variability in lake-hydrodynamics, and chemical and biological interactions. The model employed here does not assume that the IGP-predator and the IG-prey compete over a single prey species or that the populations are in equilibrium. In particular, the IGP dynamics in this study are examined under non-equilibrium conditions, where the IG-prey population shows a strong boom-bust response relative to the fish whose role is more constant, thereby capturing the natural environmental dynamics that influence the different IGP components. The explicit integration of complex networks, and non-linear and non-equilibrium modeling allows for the exploration and examination of long-term various relationships and dynamics in a system resembling the real-world food web. In the current approach, a complex model is confronted with theoretical concepts and paradigms [64]. The outcome complies with the conclusion of the simpler models that the IG-prey must be competitively superior to the IGP-predator, but uniquely shows the seasonal dynamics of an IGP-predator that is more effective at suppressing the target herbivore population (i.e., the ×8 scenario in this study), thus reducing the IG-prey and the inter-and intra-seasonal impact on the resource (herbivore population) and other related ecosystem components, such as the micro-zooplankton.

In many aspects, the model is validated both directly and indirectly. Nevertheless, there are limitations and inaccuracies in the model, producing differences between observed and simulated outcomes. For example, zooplankton dynamics do not always match empirical observations, particularly for predatory zooplankton. The model underestimates predatory zooplankton abundance; the simulated biomass is low for most of the year and the amplitude is biased towards a high value, while the observed seasonal pattern is not as clear and obvious as that seen in the model output. The herbivorous zooplankton biomass is also underestimated. However, it seems consistent that increasing its biomass will increase its predation by the predatory zooplankton and will support our conclusion that the dominant predator of the herbivorous zooplankton is the predatory zooplankton.

The differences between the simulated and observed zooplankton biomass can be attributed to: (1) the variability of the field data resulting from long-term changes that occurred in the lake ecosystem [32,55]; (2) changes in the sampling protocols, with a shift from a vertical mix sample to depth specific sampling that indicates a large degree of variation across the various months for all three zooplankton groups, thus hindering the detection of any statistically significant seasonal patterns [58]; (3) inadequate accuracy in the model at the base of the food web [39] and the absence of a self-contained model for zooplankton that accounts for the maturation of juvenile copepods in the predatory compartment. This obstacle could be eliminated by adding a stage-structured model, but at the cost of introducing additional complexity, error, and uncertainty into the model [65,66]; and (4) the averaging of spatial processes taking place in the lake in the one-dimensional modeling approach. However, the aim of the research, which was to gain a better understanding of the processes and interactions within the food web, is achieved using the current complexity of the model.

The simulation results can be further validated by future research, both in Lake Kinneret and in ecosystems where the abundance of fish or zooplankton has changed as a result of natural or anthropogenic actions (e.g., overfishing, stocking), or in ecosystems where the species composition was altered as a result of species invasion. Additionally, employing the approach presented here within a 3D hydrodynamic model would allow an exploration of the role of spatial variation in IGP interactions and is suggested as an area of future research.

## 5. Conclusions

IGP dynamics vary seasonally and influence the structure and function of aquatic food webs. When the biomass of the IGP-predator is significantly higher, like in atypical meteorological years, the effect is a shift from a bottom-up controlled ecosystem to top-down control, with seasonal exceptions leading to an increase of resource (shared prey) biomass, to extents similar to the base level (typical meteorological years). The analysis suggests that IGP dynamics should be considered in food web models, in order to more accurately capture mass transfer and trophic interactions.

## Figures and Tables

**Table 1:** Comparison of the fish (F) and zooplankton (Z) average annual predation rate (thousand ton year^−1^) and ratio of zooplankton predation to fish predation for each zooplankton group, at base level of fish (×1), at ×2 fish, and at ×8 fish. Values in parentheses indicate the relative fraction (in %) of predation pressure compared to the base level. Z1_z1_, Z1_z2_, and Z1_z3_ are predatory zooplankton predation on predatory (Z1), herbivorous (Z2), and micro (Z3) zooplankton, respectively. F_z1_, F_z2_, and F_z3_ are fish predation on predatory, herbivore, and micro zooplankton, respectively.

Variable	×1	×2	×8
Z1_z1_	12.62	6.89 (54%)	1.17 (9%)
F_z1_	2.37	3.48 (146%)	5.01 (211%)
Z1_z2_	31.81	25.32 (79%)	12.11 (38%)
F_z2_	12.83	18.57 (144%)	33.10 (258%)
Z1_z3_	0.59	0.45 (75%)	0.16 (27%)
F_z3_	0.08	0.13 (162%)	0.35 (438%)
Total Z_zi_ predation	45.02	32.66 (76%)	13.43 (30%)
Total F_zi_ predation	15.28	22.19 (145%)	38.45 (252%)
Total predation	60.3	54.84	51.9
Total predation on Z2 (Z1_z2_ + F_z2_)	44.64	43.89	45.21
Z1_z1_/F_z1_	5.32	1.98	0.23
Z1_z2_/F_z2_	2.48	1.36	0.37
Z1_z3_/F_z3_	7.7	3.4	0.45

**Table 2:** The change in the average concentration of herbivorous zooplankton (% of ×1) following changes of +50% and −50% in the parameter values. Initial parameter values are provided.

Parameter	Initial Parameter Value	−50% Change to Parameter Value	+50% Change to Parameter Value
Maximum predation rate of predatory zooplankton (g_max_)	3.03	+27%	−10%
Preference of predatory zooplankton for predatory zooplankton—self limitation (P_zk1_) *	0.15	−7%	+1%
Vulnerability of the predatory zooplankton (V_11_)	0.4	−8%	+1%
Vulnerability of the herbivorous zooplankton (V_12_)	0.5	+10%	−7%

* The preferences of predatory zooplankton to all of its prey types must sum to 1. Therefore increasing or decreasing the preference of predatory zooplankton to the predatory zooplankton (self-limitation) by ±50% requires a change in the preference to the other prey type. The preference to micro-zooplankton was modified to comply with this requirement.
